# Similar Metabolic Health in Overweight/Obese Individuals With Contrasting Metabolic Flexibility to an Oral Glucose Tolerance Test

**DOI:** 10.3389/fnut.2021.745907

**Published:** 2021-11-16

**Authors:** Rodrigo Fernández-Verdejo, Lorena Malo-Vintimilla, Juan Gutiérrez-Pino, Antonio López-Fuenzalida, Pablo Olmos, Pablo Irarrazaval, Jose E. Galgani

**Affiliations:** ^1^Carrera de Nutrición y Dietética, Departamento de Ciencias de la Salud, Facultad de Medicina, Pontificia Universidad Católica de Chile, Santiago, Chile; ^2^Laboratorio de Fisiología del Ejercicio y Metabolismo (LABFEM), Escuela de Kinesiología, Facultad de Medicina, Universidad Finis Terrae, Santiago, Chile; ^3^Departamento de Nutrición, Diabetes y Metabolismo, Facultad de Medicina, Pontificia Universidad Católica de Chile, Santiago, Chile; ^4^Carrera de Kinesiología, Departamento de Ciencias de la Salud, Facultad de Medicina, Pontificia Universidad Católica de Chile, Santiago, Chile; ^5^Disciplinary Department of Kinesiology, Faculty of Health Science, Universidad de Playa Ancha, Valparaíso, Chile; ^6^Departamento de Ingeniería Eléctrica e Instituto de Ingeniería Biológica y Médica, Escuelas de Ingeniería, Medicina y Ciencias Biológicas, Pontificia Universidad Católica de Chile, Santiago, Chile

**Keywords:** carbohydrate metabolism, insulin sensitivity, insulin resistance, lipid metabolism, fuel selection

## Abstract

**Background:** Low metabolic flexibility (MetF) may be an underlying factor for metabolic health impairment. Individuals with low MetF are thus expected to have worse metabolic health than subjects with high MetF. Therefore, we aimed to compare metabolic health in individuals with contrasting MetF to an oral glucose tolerance test (OGTT).

**Methods:** In individuals with excess body weight, we measured MetF as the change in respiratory quotient (RQ) from fasting to 1 h after ingestion of a 75-g glucose load (i.e., OGTT). Individuals were then grouped into low and high MetF (Low-MetF *n* = 12; High-MetF *n* = 13). The groups had similar body mass index, body fat, sex, age, and maximum oxygen uptake. Metabolic health markers (clinical markers, insulin sensitivity/resistance, abdominal fat, and intrahepatic fat) were compared between groups.

**Results:** Fasting glucose, triglycerides (TG), and high-density lipoprotein (HDL) were similar between groups. So were insulin sensitivity/resistance, visceral, and intrahepatic fat. Nevertheless, High-MetF individuals had higher diastolic blood pressure, a larger drop in TG concentration during the OGTT, and a borderline significant *(P* = *0.05)* higher Subcutaneous Adipose Tissue (SAT). Further, compared to Low-MetF, High-MetF individuals had an about 2-fold steeper slope for the relationship between SAT and fat mass index.

**Conclusion:** Individuals with contrasting MetF to an OGTT had similar metabolic health. Yet High-MetF appears related to enhanced circulating TG clearance and enlarged subcutaneous fat.

## Introduction

Metabolic health refers to the state of normal regulation of glucose and lipid metabolism. Impaired metabolic health appears determined by ectopic lipid accumulation and insulin resistance (1–3). Although obesity is a major risk factor for impaired metabolic health (4), a pathophysiological mechanism accounting for the progression from lean metabolically healthy to obese metabolically unhealthy remains elusive (5). Indeed, excess body weight is not sufficient to alter metabolic health, and therefore, some individuals with obesity maintain a metabolically healthy phenotype (1, 5–7). Thus, other factors should link excess body weight with poor metabolic health.

The capacity to adapt fuel oxidation to fuel availability —i.e., metabolic flexibility (MetF) (8) —has been proposed as an underlying factor of metabolic health (9, 10). An enhanced MetF potentially helps tissues to respond to a fuel oversupply that would otherwise promote ectopic lipid accumulation and insulin resistance (3, 11, 12). In agreement, previous evidence showed that men with high insulin sensitivity (vs. low) better coupled fat oxidation to elevated plasma non-esterified fatty acids (NEFA) concentration, and they also had lower (borderline) intramyocellular lipid content (13). Even so, evidence regarding the causality between MetF and metabolic health remains inconclusive, as has been extensively discussed elsewhere (8, 14). On the one hand, MetF has been assessed by different methods, thus dampening comparability among studies (8). The most used method—the euglycemic-hyperinsulinemic clamp—primarily assesses skeletal muscle MetF (15), with minor influence from organs, such as the liver (16), which is also relevant for metabolic health (17). On the other hand, different interpretations are drawn depending on the level of fuel availability. For example, compared to non-diabetic subjects, those with type-2 diabetes have lower MetF (by the clamp) when fuel availability is considered at the circulating level; but similar MetF when fuel availability is considered at tissue level (18–20).

Therefore, there is a need for a standardized method and analytical approach to understanding the role of MetF on metabolic health. A method challenging the capacity to adapt fuel oxidation to the ingestion of a nutrient excess appears methodologically and clinically relevant. Herein, we measured MetF to an oral glucose tolerance test (OGTT). This method proceeds under highly standardized, reproducible, and feasible conditions. Furthermore, an OGTT challenges the MetF of critical tissues, such as skeletal muscle and liver. We hypothesized that subjects with High-MetF to the OGTT have better metabolic health than subjects with Low-MetF to the OGTT. To test this hypothesis, we aimed to compare metabolic health in individuals with contrasting MetF, but similar excess body weight, body fat, sex, and age. Metabolic health was evaluated using the components of metabolic syndrome, along with insulin sensitivity/resistance, abdominal fat distribution, and intrahepatic fat content. In the groups with contrasting MetF to the OGTT, we additionally compared MetF to prolonged fast, a method that challenges the oxidation of endogenous lipid. This allowed us to explore whether High-MetF to exogenous glucose (the OGTT) associates with High-MetF to endogenous lipid (prolonged fast).

## Methods

### Subjects

We recruited adult women and men with overweight or obesity and stable weight (variation <2 kg in the last 3 months). The recruitment took place between July 2018 and August 2019. At the screening visit, volunteers were confirmed as healthy based on medical examination, normal blood profiles (biochemical profile, thyroid-related hormones, hemogram, and electrolytes), normal electrocardiogram, and absence of diagnosed diseases or other conditions that potentially affect energy metabolism. Volunteers were not taking medications or nutritional supplements. Some women were taking oral contraceptives, which are not expected to influence our main measurements. We excluded subjects with fasting glucose concentration >110 mg/dl. Volunteers did not exercise vigorously more than 3 times/wk or had a physically demanding job.

### Study Design

After the screening visit, subjects participated in six additional visits, ordered according to the subjects and equipment availability. The following procedures were conducted in each visit: [a] OGTT combined with indirect calorimetry to measure MetF to exogenous glucose; [b] euglycemic-hyperinsulinemic clamp to measure insulin sensitivity; [c] incremental exercise test to measure maximum oxygen uptake (VO_2_max); [d] dual-energy X-ray absorptiometry to measure body composition; [e] MRI to measure abdominal and intrahepatic fat; and [f] prolonged fast to measure MetF to endogenous lipid. For the OGTT, euglycemic-hyperinsulinemic clamp, and prolonged fast, participants were instructed to refrain from vigorous physical activity 24 h before and from tea, coffee, and other thermogenic substances 12 h before. Note that body weight changed <2 kg throughout the study.

### OGTT

After a 10–12 h overnight fast, an intravascular cannula was inserted into an antecubital vein. Participants then rested for 30 min in a supine position under thermoneutral and quiet conditions before gas exchange measurement for 20 min. During the gas exchange measurement, two 10-min apart blood samples were drawn to determine metabolic markers; the average of both samples represented the blood fasting values (“before” time-point). Then, 75 g of glucose (Trutol 75, Thermo Scientific, Waltham, MA, USA) were ingested within 5 min, and blood samples were subsequently collected at 30, 60, 90, and 120 min. Gas exchange was measured again at 40–60 and 100–120 min after glucose ingestion. MetF was considered as the change in respiratory quotient (RQ = VCO_2_/VO_2_) from fasting to 60 min after glucose ingestion (δRQ = RQ 60 min – RQ fasting). Note that δRQ was unrelated to fasting RQ in the whole group (Spearman *r* = −0.09; *P* = 0.67), therefore, we did not adjust δRQ for fasting RQ.

All gas exchange measurements were conducted with a VMax Encore 29n (SensorMedics Co., Yorba Linda, CA, USA) and corrected using high-precision mass-flow regulators (series 358; 0–2 l/min; Analyt-MTC [Müllheim, Germany]), as described (21). Metabolic rate (in kcal/d) was calculated as: [3.941 × VO_2_ (L/min) + 1.11 × VCO_2_ (L/min)] × 1,440 min. In addition, glucose-induced thermogenesis (in kcal/d × min) was computed as the incremental area under the curve by the trapezoidal method as: 2 × (kcal/d_60min_ × 30) + (kcal/d_120min_ × 30)—(kcal/d_before_ × 90).

### Euglycemic-Hyperinsulinemic Clamp

After a 10–12 h overnight fast, an intravenous catheter was inserted into an antecubital vein for infusion of insulin and a 20% glucose solution. A second intravenous catheter was inserted into an antecubital vein of the contralateral arm for blood sampling. Subjects then rested supine for 30 min, after which insulin was sequentially infused at 8 mIU/kg × min for 3 min, 4 mIU/kg × min for 2 min, and 2 mIU/kg × min for 115 min, i.e., 120 min in total. The 20% glucose solution was infused at variable flows to maintain blood glucose concentration at 85 mg/dl. Blood samples were obtained every 5 min to monitor blood glucose concentration (Biosen C-Line, Clinic/GP+, EKF-diagnostic GmbH, Magdeburg, Germany). Blood samples obtained at 100–120 min of infusion were used to calculate insulin sensitivity as the whole-body glucose disposal rate (in mg glucose/kg × min) assuming total suppression of hepatic glucose production (16). In addition, whole-body glucose disposal rate was divided by the steady-state insulin concentration × 100.

### Incremental Exercise Test

Subjects arrived at the laboratory after a 2–3 h fast and rested sitting for 15 min. Then, they exercised on a Cycle Ergometer, (CareFusion LE 200CE) following this protocol: 0 W for 2 min (warm-up), 35 W for 3 min, 70 W for 3 min, 105 W for 3 min, and 140 W for 3 min, and then the workload increased by 35 W every minute until exhaustion. Cadence was maintained at 50–60 rpm. During the test, VO_2_ and VCO_2_ were measured with a breath-by-breath gas analysis system (Ergospirometer MasterScreen CPX, JaegerTM, Germany). Heart rate was also constantly measured (H10, Polar). Subjects were considered to attain the VO_2_max if reaching at least one of these criteria (22): [a] increase in VO_2_ lower than 150 ml/min in successive workloads; [b] RQ > 1.15; [c] heart rate within 10 bpm of the maximal heart rate predicted by age (23).

### Dual-Energy X-Ray Absorptiometry and MRI

Total body fat mass was measured by dual-energy X-ray absorptiometry. Fat-free mass (FFM) was calculated as the difference between body mass and fat mass. The fat mass index was then calculated by dividing the total fat mass (kg) by the height squared (m^2^). Abdominal fat was measured by MRI using T1-weighted six-echo Dixon-type acquisitions in a Philips 1.5T (TR/TE/DTE = 30/1.3/2.1 ms; resolutio*n* = 2 × 2 × 10 mm^3^; 21 slices for liver coverage). Participants were analyzed in a supine position with the arms extended above the head. Water/fat separation was obtained using the IDEAL method proposed by Reeder et al. (24). For a more detailed description of fat imaging and its uses as a biomarker see Bray et al. (25). Abdominal fat was classified as Subcutaneous Adipose Tissue (SAT), Visceral Adipose Tissue (VAT), and intrahepatic fat. SAT and VAT were obtained from a single 10-mm slice at the lumbar region (L2–L3 level). To help in the segmentation, we combined automatic segmentation algorithms (region growing, graph cuts, and hierarchical IDEAL) implemented in a software developed by our group (26) with manual examination to exclude intermuscular and paravertebral adipose tissue from the VAT area. All images were inspected to corroborate the quality of segmentation.

### Prolonged Fast

After a 10–12 h overnight fast, an intravenous catheter was inserted into an antecubital vein for blood sampling. Subjects then rested supine for 30 min, and gas exchange was measured and corrected as described for the OGTT (section OGTT). Two blood samples were obtained 10-min apart during the gas exchange measurement to determine metabolic markers; the average of both samples represented the blood fasting values (“before” time-point). The intravenous catheter was then removed. Next, subjects stayed sitting or lying in the laboratory for 7 h and were allowed to read, watch TV, use a computer or smartphone, and use the restroom. Water was provided *ad libitum*, but no other beverage or food was consumed. After, an intravenous catheter was inserted into an antecubital vein of the other arm. Subjects rested supine for 30 min, and gas exchange was measured again. Two blood samples were obtained 10-min apart during the gas exchange measurement to determine metabolic markers; the average of both samples represented the values at the end of prolonged fast (“after” time-point).

### Blood Analyses and Markers of Metabolic Health

Serum was used to determine insulin concentrations by chemiluminescence, along with triglycerides (TG), total cholesterol, and high-density lipoprotein-cholesterol (HDL) concentrations by dry chemistry. Whole-blood glucose and lactate were measured by an electrochemical method (Biosen C-Line device). Plasma glucose was measured by the glucose oxidase method. Colorimetric assay kits were used to measure serum concentrations of NEFA (NEFA-HR[2], Wako Diagnostics, Richmond, VA, USA), glycerol (MAK117, Sigma-Aldrich, St. Louis, MO, USA), and β-hydroxybutyrate (βOHB; #700190, Cayman Chemical, Ann Arbor, MI, USA).

From the blood samples of the OGTT, several health markers were calculated. The Homeostasis Model Assessment of Insulin Resistance (HOMA-IR) (27) was calculated from fasting samples as: [plasma glucose (mmol/L) × serum insulin (mIU/L)]/22.5. The Matsuda index (28) was calculated from fasting and postprandial concentrations of glucose and insulin. Adipose tissue insulin resistance (Adipo-IR index) (29) was calculated from fasting samples as: NEFA (mmol/L) × insulin (pmol/L). The insulinogenic index (30) was calculated as: (serum insulin (mIU/L) [30 min – fasting])/(plasma glucose (mg/dl) [30 min – fasting]). Finally, NEFA suppression was calculated as: NEFA nadir (mmol/L)/NEFA fasting (mmol/L). In all cases, fasting samples are the same as the “before” time-point described for the OGTT (section OGTT).

From measurements at the screening visit, subjects were considered to have metabolic syndrome if manifested ≥3 of the following disturbances (6): [a] waist circumference (WC) ≥91 cm in men or ≥83 cm in women; [b] TG ≥150 mg/dl; [c] HDL <40 mg/dl in men or <50 mg/dl in women; [d] diastolic blood pressure ≥85 mmHg or systolic blood pressure ≥130 mmHg; and [e] glucose ≥100 mg/dl. Furthermore, we calculated a metabolic syndrome z-score, as previously done (31). The z-score indicates the severity of the syndrome by integrating its five components in a continuous variable. This is a better index of severity than just adding the number of disturbances because the same disturbance may have different severity, e.g., TG of 151 vs. 300 mg/dl. The higher the z-score, the higher the severity of the syndrome. We used the SDs of each metabolic syndrome component obtained from the National Health Survey of Chile 2016–2017 (32) to calculate the z-score: in males = (40 – HDL)/12.1 + (TG – 150)/98.5 + (glucose – 100)/23 + (WC – 91)/12 + (mean arterial pressure [MAP] – 100)/11; in females = (50 – HDL)/13.1 + (TG – 150)/98.5 + (glucose – 100)/23 + (WC – 83)/13.6 + (MAP – 100)/11. For calculations, HDL, TG, and glucose were expressed in mg/dl, WC in cm, and MAP in mmHg. MAP was calculated as: diastolic blood pressure + (systolic blood pressure – diastolic blood pressure)/3.

### Sample Size Calculation

We considered the HOMA-IR and Matsuda index as the health outcomes for sample size calculation. Considering their inter-individual variability (33), to detect differences in HOMA-IR of ≥1.3 and in Matsuda index of ≥2.3 between two groups, we needed 12–13 subjects per group (80% power, 5% type I error). Thus, we recruited 25 individuals that were subsequently divided into low (Low-MetF, *n* = 12) or high (High-MetF, *n* = 13) MetF by the median value of δRQ in the OGTT. The median δRQ was 0.036, representing the ~20th percentile from a previous study in healthy individuals (body mass index 20–35 kg/m^2^) assessed with similar instrumentation and conditions (33). There were no differences in sex, age, body mass index, and VO_2_max between groups (Table 1).

**Table 1:** General characteristics of subjects.

	**Low-MetF**	**High-MetF**	***P*** -value^*^
Females/Males (*n*)	8/4	8/5	0.99
Age (years)	29.9 (25.8–39.5)	28.6 (27.3–34.5)	0.77
Body weight (kg)	82.6 (72.6–86.1)	86.5 (79.4–96.9)	0.22
Body mass index (kg/m^2^)	29.6 (28.2–32.2)	30.5 (28.9–33.1)	0.41
Maximum oxygen uptake (mL/kg × min)	21.9 (19.9–24.9)	28.2 (23.0–30.2)	0.15

*Data are median (interquartile range) or the number of individuals.*

* *Wilcoxon test with two-sided t approximation or Fisher test*.

### Statistical Analyses

Results are presented as median [interquartile range] or proportions. Analyses were performed using SAS version 9.4 (SAS Institute, Cary, NC, USA). Proportions and distributions between groups were analyzed by Fisher and Wilcoxon (with two-sided *t* approximation) tests, respectively. Raw values were log-transformed or ranked as needed to conduct repeated-measures ANOVA for assessing the effects of group (Low-MetF, High-MetF), time (before and after the OGTT, or before and after the prolonged fast), and group × time interaction. In case of significant interaction, Tukey *post-hoc* test was conducted to compare before vs. after values within the same group or Low-MetF vs. High-MetF values at the same time-point. Analysis of covariance (ANCOVA) was used to compare regression equations between groups. A *P* < 0.05 was considered statistically significant.

## Results

### MetF and Metabolic Responses to the OGTT

**Figure 1:**
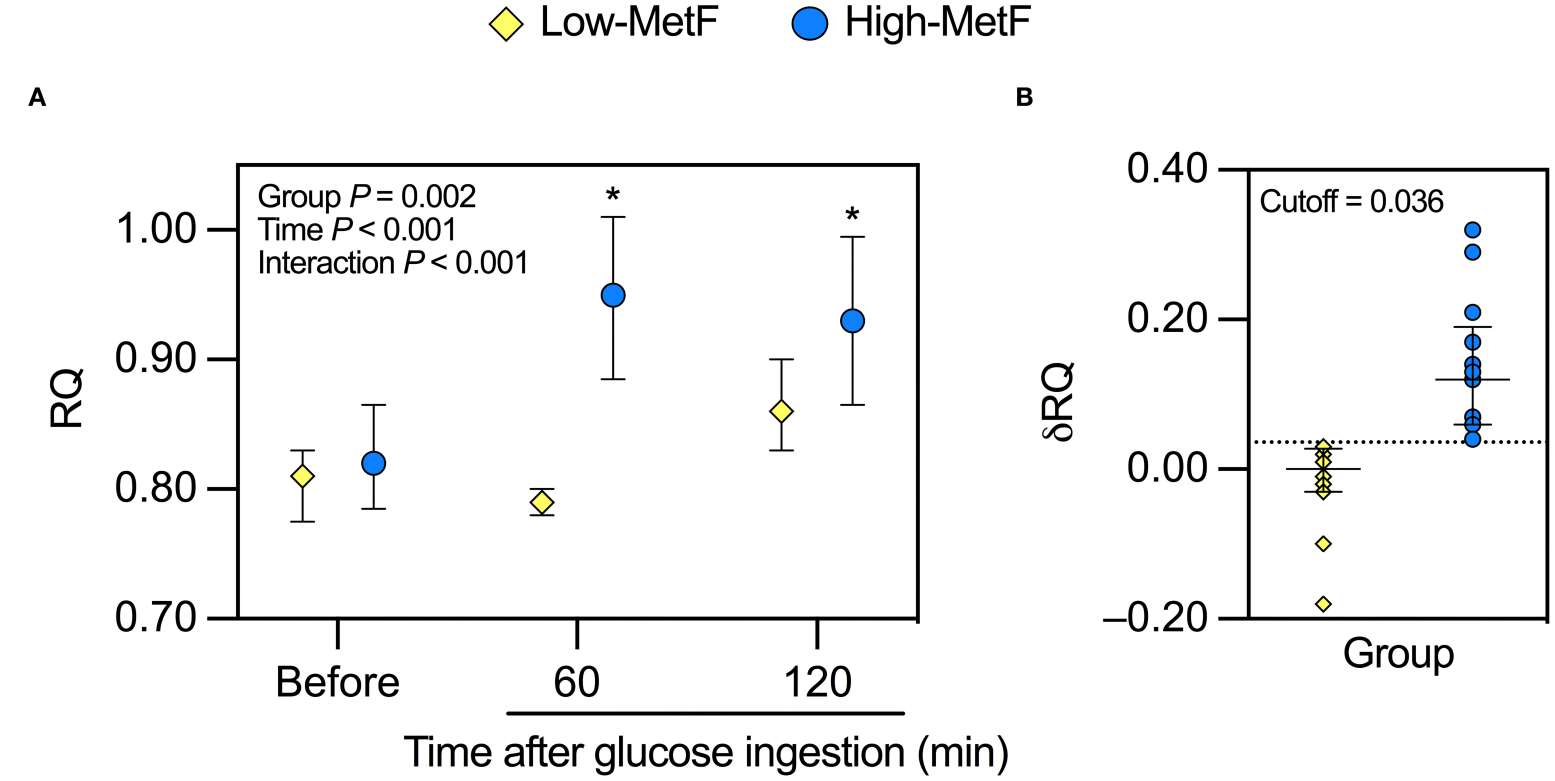
Response of the respiratory quotient (RQ) to a 75-g oral glucose tolerance test in individuals with low (Low-MetF) and high (High-MetF) metabolic flexibility. **(A)** RQ before, 60 after, and 120 min after glucose ingestion. **(B)** Change in RQ (δRQ) from before to 60 min after glucose ingestion (RQ at 60 min – RQ before); a δRQ of 0.036 was the cutoff to divide subjects according to MetF. Data are median and interquartile ranges. Data were analyzed by repeated-measures ANOVA and Tukey *post-hoc*. *P < 0.05 vs. before within the same group.

Overnight fasting RQ was similar in the High-MetF and Low-MetF groups (0.82 [0.79–0.84] and 0.81 [0.78–0.83], respectively; *P* = 0.99) and increased to a different extent after glucose ingestion by design (Figure 1A). Thus, MetF in response to OGTT (i.e., δRQ over the first hour) was 0.12 [0.06–0.17] in the High-MetF and 0.00 [−0.03–0.03]) in the Low-MetF groups (Figure 1B). In turn, the Low-MetF group achieved similar RQ values as individuals with High-MetF after 2 h of glucose ingestion.

**Figure 2:**
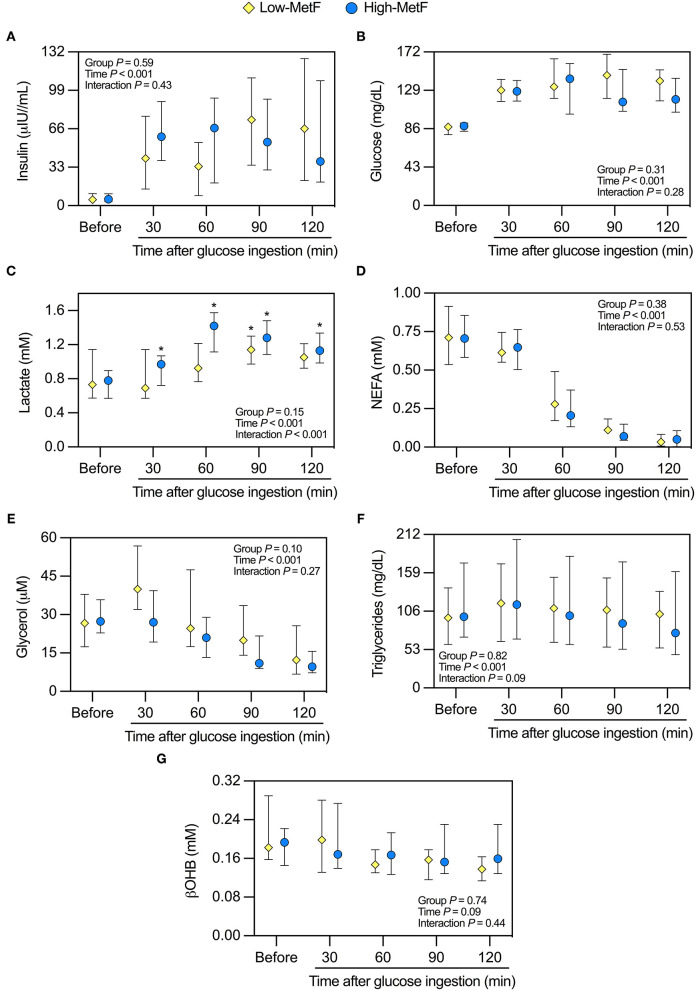
Response of circulating markers to a 75-g oral glucose tolerance test in individuals with low (Low-MetF) and high (High-MetF) metabolic flexibility. Concentrations were measured before and at various time-points after glucose ingestion for **(A)** insulin, **(B)** glucose, **(C)** lactate, **(D)** non-esterified fatty acids [NEFA], **(E)** glycerol, **(F)** triglycerides, and **(G)** β-hydroxybutyrate [βOHB]. Data are median and interquartile ranges. Data were analyzed by repeated-measures ANOVA and Tukey *post-hoc*. *P < 0.05 vs. before within the same group.

We additionally explored the metabolic responses to the OGTT. Glucose-induced thermogenesis was similar in High-MetF and Low-MetF groups (22,793 [12,264–29,618] and 13,200 [7,500–18,498] kcal/d × min, respectively; *P* = 0.13). In both groups, circulating levels of insulin, glucose, NEFA, and glycerol were changed similarly after the OGTT, with serum βOHB achieving borderline significance (*P* = 0.09; Figure 2). As for lactate, there was a group × time interaction. In the High-MetF group, lactate concentration remained higher than fasting values over the entire post-glucose ingestion period. In turn, lactate was only increased after 90 min of glucose ingestion in the Low-MetF group (Figure 2C). A borderline significant (*P* = 0.09) group × time interaction was noted in serum TG (Figure 2E). Further analyses showed a larger drop in serum TG concentration (TG 120 min–TG before) in the High-MetF vs. Low-MetF group (−26 [−21 to −13] vs. −4 [−8 to 6] mg/dl, respectively; *P* = 0.03). NEFA suppression was similar in the High-MetF and Low-MetF groups (0.07 [0.00–0.10] and 0.06 [0.01–0.09], respectively; *P* = 0.98).

### Metabolic Health in Low-MetF vs. High-MetF Groups

#### Clinical Markers

During the OGTT, both glucose tolerance (Figure 2B) and the insulinogenic index were similar in the High-MetF and Low-MetF groups (1.04 [0.67–2.73] and 0.79 [0.38–1.40] (μIU/ml)/(mg/dl), respectively; *P* = 0.23). So were the markers of metabolic syndrome assessed at the screening visit, except for a slightly higher diastolic blood pressure in individuals with High-MetF vs. Low-MetF (Table 2). In the same line, both groups had similar metabolic syndrome z-scores and the proportion of subjects with metabolic syndrome (Table 2).

**Table 2:** Metabolic health in individuals with low (Low-MetF) and high (High-MetF) metabolic flexibility.

	**Low-MetF (*n =* 12)**	**High-MetF (*n =* 13)**	***P*** -value^*^
**Clinical markers**			
Waist circumference (cm)	96 (89–101)	98 (93–106)	0.25
Systolic blood pressure (mmHg)	122 (115–129)	126 (124–130)	0.19
Diastolic blood pressure (mmHg)	75 (68–77)	80 (73–86)	0.04
Fasting glucose (mg/dL)	90 (86–98)	92 (85–98)	0.94
Fasting triglycerides (mg/dL)	107 (97–123)	120 (76–150)	0.89
Fasting HDL cholesterol (mg/dL)	42 (39–59)	45 (35–48)	0.94
Metabolic syndrome z-score	−0.40 (−2.77 to 0.12)	0.80 (−1.79 to 1.68)	0.23
Metabolic syndrome diagnosis (*n*)	3	8	0.11
**Body fat mass and distribution**			
Body fat mass (%)	46.3 (41.2–49.8)	46.1 (41.8–50.7)	0.94
Fat mass index (kg/m^2^)	13.4 (12.1–16.5)	14.0 (12.8–16.2)	0.46
Visceral abdominal tissue [VAT] (cm^3^)	120 (94–168)	123 (104–161)	0.98
Subcutaneous abdominal tissue [SAT] (cm^3^)	214 (130–223)	238 (220–281)	0.05
SAT/(SAT + VAT)	0.63 (0.52–0.69)	0.68 (0.61–0.72)	0.11
Intrahepatic fat (%)	6.5 (5.2–13.2)	7.9 (5.3–15.2)	0.83
**Insulin sensitivity/resistance**			
HOMA-IR^†^	1.06 (0.73–2.31)	1.20 (0.94–2.21)	0.73
Matsuda^†^	5.8 (3.9–6.7)	4.5 (3.5–8.1)	0.81
GDR by clamp (mg/kg × min)	5.6 (5.0–11.4)	7.4 (5.1–9.6)	0.86
GDR by clamp (mg/kg × min × IU insulin)	4.9 (2.7–5.7)	7.2 (3.1–8.0)	0.58
Adipose tissue insulin resistance index^†^	20.8 (15.0–43.1)	20.6 (16.8–44.8)	0.89

*Data are median (interquartile range) or number of individuals.*

* *Wilcoxon test with two-sided t approximation or Fisher test;*

† *From oral-glucose tolerance test; GDR, whole-body glucose disposal rate*.

#### Body Fat Mass and Distribution

**Figure 3:**
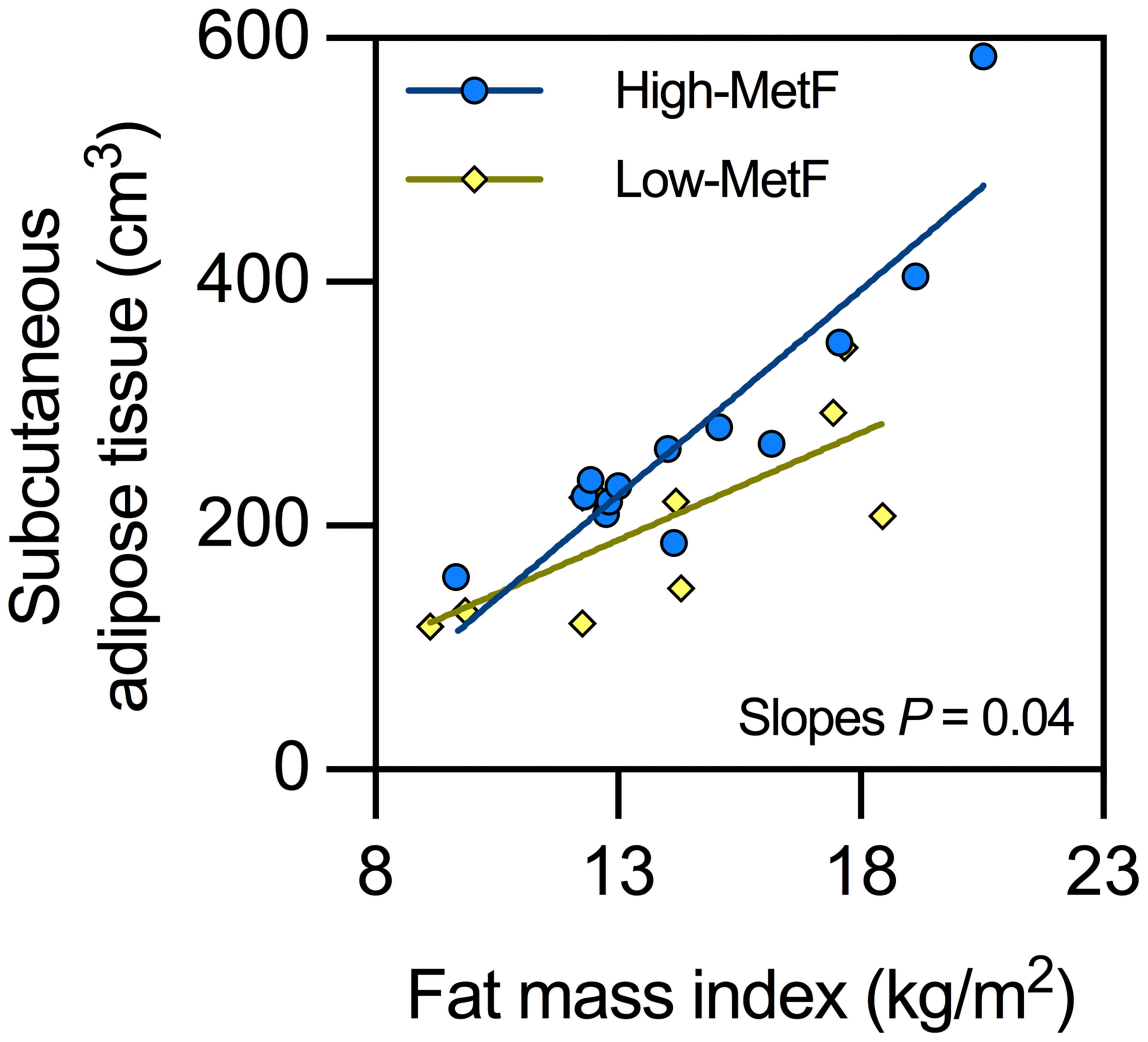
Relationship between subcutaneous adipose tissue and fat mass index in individuals with low (Low-MetF) and high (High-MetF) metabolic flexibility. Slopes were compared by ANCOVA.

Total body fat mass was similar between groups (Table 2), as expected from their similar sex, age, and body mass index. VAT was also similar, whereas SAT showed a borderline significant (*P* = 0.05) higher value in High-MetF vs. Low-MetF (Table 2). Notably, analyses of the relationship between SAT and fat mass index showed a steeper slope [SE] in the High-MetF vs. Low-MetF group (33.7 [4.7] vs. 17.6 [5.6] cm^3^ per unit of the fat mass index, respectively; *P* = 0.04; Figure 3). Intrahepatic fat content was similar between groups (Table 2).

#### Insulin Sensitivity/Resistance

Neither HOMA-IR [a marker of hepatic insulin sensitivity (27)], Matsuda index [a marker integrating hepatic and skeletal muscle insulin sensitivity (34)], glucose disposal rate by the clamp [determined mainly by skeletal muscle insulin sensitivity (15)], nor Adipo-IR index [a marker of adipose tissue insulin sensitivity (29)] was different between groups (Table 2).

### MetF and Metabolic Response to Prolonged Fast in Low-MetF vs. High-MetF Groups

Overnight fasting RQ was similar in the High-MetF and Low-MetF groups (0.81 [0.79–0.85] and 0.80 [0.78–0.84], respectively; *P* = 0.87), and decreased to a similar extent after prolonged fast (Supplementary Figure 1A). Thus, δRQ to prolonged fast –i.e., MetF to prolonged fast– was similar in the High-MetF and Low-MetF groups (−0.03 [−0.07 to −0.02] and −0.03 [−0.08 to −0.01], respectively; *P* = 0.73). Finally, metabolic rate was increased similarly in the High-MetF and Low-MetF groups (98 [36–205] and 52 [21–92] kcal/d, respectively; *P* = 0.20), and blood insulin, glucose, lactate, NEFA, glycerol, TG, and βOHB concentrations also responded similarly in both groups (Supplementary Figures 1B–H).

## Discussion

Metabolic flexibility represents the capacity to adapt fuel oxidation to fuel availability and is considered a component of the healthy metabolic phenotype (8, 35). We thus hypothesized that subjects with High-MetF would have better metabolic health than individuals with Low-MetF. Nevertheless, we observed that individuals with contrasting MetF to an OGTT had similar metabolic health, assessed by the clinical markers of metabolic syndrome, insulin sensitivity/resistance indexes, and intrahepatic fat content. The only differences between groups were that individuals with High-MetF had a slightly (~5 mmHg) higher diastolic blood pressure, a borderline significantly larger SAT, a faster increase in lactate during the OGTT, and a higher TG clearance during the OGTT. These results suggest that MetF to an OGTT does not discriminate for metabolic health in groups homogenous for excess body weight, body composition, VO_2_max, and other confounding factors. At the same time, our results point toward an association between MetF and SAT that has been scarcely explored.

There are several methods to assess MetF. Each method challenges different tissues for their capacity to adapt the oxidation to the availability of different substrates and sources (8). For example, the hyperinsulinemic-euglycemic clamp mostly challenges skeletal muscle for exogenous glucose oxidation. Whereas, prolonged fast challenges both the liver and skeletal muscle for endogenous NEFA oxidation. In lean, healthy men, we have previously observed that both measures of MetF are directly associated, suggesting concordance in MetF in these organs (36). Herein, we reasoned that a method approaching hepatic MetF to exogenous nutrient excess maybe relevant. This is because among individuals with obesity, those with low (vs. high) hepatic fat content seem characterized by an enhanced capacity to handle dietary nutrient excess (7). Indeed, after gaining weight due to dietary excess, these individuals accumulated less hepatic fat and preserved their insulin sensitivity (7). Therefore, we propose that the extent to which RQ changes over the first hour of an OGTT (where the organism is exposed to a ~4–5 times energy excess) represents a valid index of MetF. Thus, individuals with the higher increase in δRQ were considered as having High-MetF, i.e., a higher capacity to adapt exogenous glucose oxidation to glucose availability. Note that these individuals also showed faster increases in blood lactate concentration, thus suggesting an enhanced non-oxidative glucose utilization as well. In contrast, individuals with Low-MetF manifested delayed increases in oxidative and non-oxidative glucose utilization. This last observation highlights an impaired capacity to switch between metabolic substrates, which has been considered a landmark of impaired MetF (37). Notably, we did not detect differences in metabolic health in individuals with contrasting MetF to an OGTT. Moreover, both groups of contrasting MetF to the OGTT displayed similar MetF to prolonged fast. These findings highlight that the factors that determine MetF depend on how MetF is measured.

Previous studies have suggested that individuals with metabolic syndrome have impaired MetF. San-Millán and Brooks (38) measured MetF as fat oxidation during an incremental exercise until exhaustion. Subjects with metabolic syndrome had lower MetF compared to moderately active individuals and professional athletes (38). From these data, we calculated that the δRQ (RQ at exhaustion—RQ at the lowest intensity) was the lowest in subjects with metabolic syndrome (8), thus supporting their impaired MetF. In another study, MetF to a high-fat meal was compared in healthy individuals and individuals with metabolic syndrome (39). The RQ dropped after meal ingestion, reaching a nadir at 30 min in healthy individuals and at 60 min in individuals with metabolic syndrome (39). These results were interpreted as indicative of impaired MetF in metabolic syndrome. Using euglycemic-hyperinsulinemic clamp (9) or moderate-intensity exercise (13), impaired MetF has been also associated with low insulin sensitivity. In contrast, in our current study, subjects with contrasting MetF to an OGTT had essentially no difference in the components of metabolic syndrome nor markers of insulin sensitivity/resistance. The presence of confounding variables may explain the discrepancy between studies. Note that our Low-MetF and High-MetF groups were similar in many putative confounding variables –e.g., body mass index, fat mass, VAT, VO_2_max–, which was not the case in previous studies (9, 38, 39). Differences in the method to assess and analyze MetF may also explain the discrepancy. For example, the classical association between insulin sensitivity and MetF to a euglycemic-hyperinsulinemic clamp occurs because δRQ depends on the glucose available for oxidation within tissues. Thus, the association often disappears after controlling for glucose disposal rate (18–20). In turn, during an OGTT, δRQ will depend on every factor from the gastrointestinal to the mitochondrial function (8). Thus, an association between MetF during an OGTT and insulin sensitivity/resistance is less apparent, as observed in our current study.

Consequently, if MetF truly influences metabolic health –with the liver as a relevant organ— this would not be detected with an OGTT, even though the liver is the first organ reached by absorbed glucose and a relevant glucose disposal site (40, 41). Alternatively, MetF to an OGTT might be an early marker for the development of metabolic disturbances. A previous study showed impairments in MetF to a mixed meal after 21 days of bed rest; but notably, the effect occurred before alterations in fasting glucose, fasting TG, or oral glucose tolerance (10). This may explain why our —yet healthy— subjects with contrasting MetF to an OGTT had similar metabolic health. Note that the slightly higher diastolic blood pressure in the High-MetF group is, however, difficult to explain. Previous evidence has suggested that obesity influences elevations in blood pressure, independent of other metabolic syndrome components (42). Nevertheless, the reason for higher diastolic blood pressure in individuals with a supposedly better metabolic profile (i.e., higher MetF) is unknown. Future studies should explore whether this is a random or consistent observation.

Limited focus has been put on a possible association between MetF and adipose tissue function. Indeed, white adipose tissue and its lipogenic capacity are critical factors determining nutrient handling capacity and metabolic health. Several studies have observed that individuals with abnormal metabolic health manifest decreased expression of genes involved in glucose uptake and lipogenesis in adipose tissue (7, 43–47). Furthermore, previous evidence suggests that the lipogenic capacity of white adipose tissue may also influence MetF. A mouse model of adipose tissue-specific PPARγ2 deficiency showed slow lipid storage in adipose tissue along with low MetF in the fed state (48). Low MetF appeared explained by an increased NEFA flux to ectopic tissues, which triggered compensatory lipid oxidation to clear lipid excess. The balance between fatty acid uptake and oxidation in ectopic tissues will finally determine the extent of fatty acid storage in those tissues. Our current results show larger SAT (for a similar total body fat content) in individuals with High-MetF vs. Low-MetF, which is consistent with the observations in mice. Perhaps, that large SAT denotes an increased capacity to store lipids that results in an enhanced capacity to oxidize carbohydrates after glucose ingestion. Notably, High-MetF individuals (vs. Low-MetF) also showed a larger drop in serum TG and a similar NEFA suppression after glucose ingestion. These latter data suggest enhanced circulating TG hydrolytic rate and preserved NEFA uptake/release in adipose tissue. Evidence indicating that a large SAT is associated with a lower frequency of elevated circulating TG concentration in humans (49) suggests that High-MetF and high SAT are related to better metabolic health.

Our study has some limitations that are worth recognizing. First, the sample size of groups was rather small, which may have precluded us from identifying differences in some parameters. However, note that based on our calculations, the sample size was adequate to detect differences in two clinical markers of insulin sensitivity/resistance. Second, the study was neither powered to test for an effect of sex nor a possible effect of oral contraceptives in women. We just ensured that the groups were balanced by sex to avoid its confounding effect. Third, contrasting MetF between groups may not be stable in time because fasting RQ often affects MetF assessed by the δRQ (20, 50, 51). Volunteers in this study maintained steady body weight, thus suggesting an energy balance in equilibrium, the main factor affecting fasting RQ (52–54). We observed a fasting RQ consistent with the consumption of mixed diets (54) that remained similar across testing days, with similar intra-individual variance between groups (*P* = 0.70, *F*-test; data not shown). These conditions and observations suggest that fasting RQ was a stable phenotype with no confounding influence on our measure of MetF.

In conclusion, our cross-sectional comparison of individuals with contrasting MetF to an OGTT does not support that high MetF associates with better metabolic health. In turn, individuals with high MetF appear to show enlarged SAT and enhanced circulating TG clearance. Whether high MetF is a trait associated with enhanced adipose tissue function, which protects from the development of abnormal metabolic health, remains elusive. This represents a potential area of future research. To accomplish that goal, consensus on how to approach MetF will be of utmost importance.

## Data Availability Statement

The raw data supporting the conclusions of this article will be made available by the authors, without undue reservation.

## Ethics Statement

The studies involving human participants were reviewed and approved by the Ethical Board at Pontificia Universidad Católica de Chile (#160809006). The patients/participants provided their written informed consent to participate in this study.

## Author Contributions

RF-V conceived the study, acquired the data, processed the data, and interpreted the data. LM-V, JG-P, and AL-F acquired the data and processed the data. PO and PI conceived the study and acquired the data. JG conceived the study, acquired the data, analyzed the data, interpreted the data, and drafted the manuscript. All authors critically revised the manuscript and approved the final version.

## Funding

The work was funded by ANID/CONICYT FONDECYT Iniciación (11180361 to RF-V), and ANID/CONICYT FONDECYT Regular (1170117 to JG).

## Conflict of Interest

The authors declare that the research was conducted in the absence of any commercial or financial relationships that could be construed as a potential conflict of interest.

## Publisher's Note

All claims expressed in this article are solely those of the authors and do not necessarily represent those of their affiliated organizations, or those of the publisher, the editors and the reviewers. Any product that may be evaluated in this article, or claim that may be made by its manufacturer, is not guaranteed or endorsed by the publisher.

## References

[1] McGarry JD. Banting lecture 2001: dysregulation of fatty acid metabolism in the etiology of type 2 diabetes. Diabetes. (2002) 51:7–18. 10.2337/diabetes.51.1.7 11756317

[2] Moro C Galgani JE Luu L Pasarica M Mairal A Bajpeyi S. Influence of gender, obesity, and muscle lipase activity on intramyocellular lipids in sedentary individuals. J Clin Endocrinol Metab. (2009) 94:3440–7. 10.1210/jc.2009-0053 19531593 PMC2741707

[3] Petersen MC Shulman GI. Mechanisms of insulin action and insulin resistance. Physiol Rev. (2018) 98:2133–223. 10.1152/physrev.00063.2017 30067154 PMC6170977

[4] Czech MP. Insulin action and resistance in obesity and type 2 diabetes. Nat Med. (2017) 23:804–14. 10.1038/nm.4350 28697184 PMC6048953

[5] Stefan N. Causes, consequences, and treatment of metabolically unhealthy fat distribution. Lancet Diabetes Endocrinol. (2020) 8:616–27. 10.1016/S2213-8587(20)30110-8 32559477

[6] Fernández-Verdejo R Moya-Osorio JL Fuentes-López E Galgani JE. Metabolic health and its association with lifestyle habits according to nutritional status in Chile: A cross-sectional study from the National Health Survey 2016-2017. PLoS ONE. (2020) 15:e0236451. 10.1371/journal.pone.0236451 32697789 PMC7375524

[7] Fabbrini E Yoshino J Yoshino M Magkos F Tiemann Luecking C Samovski D. Metabolically normal obese people are protected from adverse effects following weight gain. J Clin Invest. (2015) 125:787–95. 10.1172/JCI78425 25555214 PMC4319438

[8] Galgani JE Fernández-Verdejo R. Pathophysiological role of metabolic flexibility on metabolic health. Obes Rev. (2021) 22:e13131. 10.1111/obr.13131 32815226

[9] Kelley DE Goodpaster B Wing RR Simoneau JA. Skeletal muscle fatty acid metabolism in association with insulin resistance, obesity, and weight loss. Am J Physiol. (1999) 277:E1130–41. 10.1152/ajpendo.1999.277.6.E1130 10600804

[10] Rudwill F O'Gorman D Lefai E Chery I Zahariev A Normand S. Metabolic inflexibility is an early marker of bed-rest-induced glucose intolerance even when fat mass is stable. J Clin Endocrinol Metab. (2018) 103:1910–20. 10.1210/jc.2017-02267 29546280 PMC7263792

[11] Bosy-Westphal A Braun W Albrecht V Müller MJ. Determinants of ectopic liver fat in metabolic disease. Eur J Clin Nutr. (2019) 73:209–14. 10.1038/s41430-018-0323-7 30323174

[12] Fletcher JA Deja S Satapati S Fu X Burgess SC Browning JD. Impaired ketogenesis and increased acetyl-CoA oxidation promote hyperglycemia in human fatty liver. JCI Insight. (2019) 4:e127737. 10.1172/jci.insight.127737 31012869 PMC6629163

[13] Fernández-Verdejo R Bajpeyi S Ravussin E Galgani JE. Metabolic flexibility to lipid availability during exercise is enhanced in individuals with high insulin sensitivity. Am J Physiol Endocrinol Metab. (2018) 315:E715–22. 10.1152/ajpendo.00126.2018 29870678 PMC6230709

[14] Tsilingiris D Tzeravini E Koliaki C Dalamaga M Kokkinos A. the role of mitochondrial adaptation and metabolic flexibility in the pathophysiology of obesity and insulin resistance: an updated overview. Curr Obes Rep. (2021) 10:191–213. 10.1007/s13679-021-00434-0 33840072

[15] Baron AD Brechtel G Wallace P Edelman S V. Rates and tissue sites of non-insulin- and insulin-mediated glucose uptake in humans. Am J Physiol. (1988) 255:E769–74. 10.1152/ajpendo.1988.255.6.E769 3059816

[16] Campbell PJ Mandarino LJ Gerich JE. Quantification of the relative impairment in actions of insulin on hepatic glucose production and peripheral glucose uptake in non-insulin-dependent diabetes mellitus. Metabolism. (1988) 37:15–21. 10.1016/0026-0495(88)90023-6 3275857

[17] Fabbrini E Magkos F Mohammed BS Pietka T Abumrad NA Patterson BW. Intrahepatic fat, not visceral fat, is linked with metabolic complications of obesity. Proc Natl Acad Sci USA. (2009) 106:15430–5. 10.1073/pnas.0904944106 19706383 PMC2741268

[18] van de Weijer T Sparks LM Phielix E Meex RC van Herpen NA Hesselink MKC. Relationships between mitochondrial function and metabolic flexibility in type 2 diabetes mellitus. PLoS ONE. (2013) 8:e51648. 10.1371/journal.pone.0051648 23418416 PMC3572106

[19] Kelley DE Mandarino LJ. Hyperglycemia normalizes insulin-stimulated skeletal muscle glucose oxidation and storage in noninsulin-dependent diabetes mellitus. J Clin Invest. (1990) 86:1999–2007. 10.1172/JCI114935 2123890 PMC329837

[20] Galgani JE Heilbronn LK Azuma K Kelley DE Albu JB Pi-Sunyer X. Metabolic flexibility in response to glucose is not impaired in people with type 2 diabetes after controlling for glucose disposal rate. Diabetes. (2008) 57:841–5. 10.2337/db08-0043 18285553 PMC2756651

[21] Galgani JE Castro-Sepulveda MA. Influence of a gas exchange correction procedure on resting metabolic rate and respiratory quotient in humans. Obesity. (2017) 25:1941–7. 10.1002/oby.21981 28924987

[22] Howley ET Bassett DR Welch HG. Criteria for maximal oxygen uptake: review and commentary. Med Sci Sports Exerc. (1995) 27:1292–301. 10.1249/00005768-199509000-00009 8531628

[23] Jones NL Makrides L Hitchcock C Chypchar T McCartney N. Normal standards for an incremental progressive cycle ergometer test. Am Rev Respir Dis. (1985) 131:700–8. 3923878 10.1164/arrd.1985.131.5.700

[24] Reeder SB Wen Z Yu H Pineda AR Gold GE Markl M. Multicoil Dixon chemical species separation with an iterative least-squares estimation method. Magn Reson Med. (2004) 51:35–45. 10.1002/mrm.10675 14705043

[25] Bray TJ Chouhan MD Punwani S Bainbridge A Hall-Craggs MA. Fat fraction mapping using magnetic resonance imaging: insight into pathophysiology. Br J Radiol. (2017) 91:20170344. 10.1259/bjr.20170344 28936896 PMC6223159

[26] Wiens CN Arrieta C Osorio I Ratliff B Colgan TJ McMillan AB. Longitudinal assessment of visceral and subcutaneous adipose tissue in obese patients undergoing weight loss surgery. In: Proceedings of the Annual Meeting of the International Society of Magnetic Resonance in Medicine, (Honolulu, HI) 3450 (2017).

[27] Matthews DR Hosker JP Rudenski AS Naylor BA Treacher DF Turner RC. Homeostasis model assessment: insulin resistance and beta-cell function from fasting plasma glucose and insulin concentrations in man. Diabetologia. (1985) 28:412–9. 10.1007/BF00280883 3899825

[28] Matsuda M DeFronzo RA. Insulin sensitivity indices obtained from oral glucose tolerance testing: comparison with the euglycemic insulin clamp. Diabetes Care. (1999) 22:1462–70. 10.2337/diacare.22.9.1462 10480510

[29] Søndergaard E Espinosa De Ycaza AE Morgan-Bathke M Jensen MD. How to measure adipose tissue insulin sensitivity. J Clin Endocrinol Metab. (2017) 102:1193–99. 10.1210/jc.2017-00047 28323973 PMC5460729

[30] Tura A Kautzky-Willer A Pacini G. Insulinogenic indices from insulin and C-peptide: comparison of beta-cell function from OGTT and IVGTT. Diabetes Res Clin Pract. (2006) 72:298–301. 10.1016/j.diabres.2005.10.005 16325298

[31] Johnson JL Slentz CA Houmard JA Samsa GP Duscha BD Aiken LB. Exercise training amount and intensity effects on metabolic syndrome (from Studies of a Targeted Risk Reduction Intervention through Defined Exercise). Am J Cardiol. (2007) 100:1759–66. 10.1016/j.amjcard.2007.07.027 18082522 PMC2190779

[32] Departamento de Epidemiologinisterio de Salud de Chile. Encuesta Nacional de Salud 2016-2017. Informe Final. Santiago: Gobierno de Chile (2017).

[33] Galgani JE Gómez C Mizgier ML Gutierrez J Santos JL Olmos P. Assessment of the role of metabolic determinants on the relationship between insulin sensitivity and secretion. PLoS One. (2016) 11:e0168352. 10.1371/journal.pone.0168352 28002466 PMC5176173

[34] Abdul-Ghani MA Matsuda M Balas B DeFronzo RA. Muscle and liver insulin resistance indexes derived from the oral glucose tolerance test. Diabetes Care. (2007) 30:89–94. 10.2337/dc06-1519 17192339

[35] Rynders CA Blanc S DeJong N Bessesen DH Bergouignan A. Sedentary behaviour is a key determinant of metabolic inflexibility. J Physiol. (2018) 596:1319–30. 10.1113/JP273282 28543022 PMC5899985

[36] Fernández-Verdejo R Castro-Sepulveda M Gutiérrez-Pino J Malo-Vintimilla L López-Fuenzalida A Olmos P. Direct relationship between metabolic flexibility measured during glucose clamp and prolonged fast in men. Obesity. (2020) 28:1110–6. 10.1002/oby.22783 32369268

[37] Kelley DE Mandarino LJ. Fuel selection in human skeletal muscle in insulin resistance: a reexamination. Diabetes. (2000) 49:677–83. 10.2337/diabetes.49.5.677 10905472

[38] San-Millán I Brooks GA. Assessment of metabolic flexibility by means of measuring blood lactate, fat, and carbohydrate oxidation responses to exercise in professional endurance athletes and less-fit individuals. Sports Med. (2018) 48:467–79. 10.1007/s40279-017-0751-x 28623613

[39] Kardinaal AFM van Erk MJ Dutman AE Stroeve JHM van de Steeg E Bijlsma S. Quantifying phenotypic flexibility as the response to a high-fat challenge test in different states of metabolic health. FASEB J. (2015) 29:4600–13. 10.1096/fj.14-269852 26198450

[40] Ferrannini E Bjorkman O Reichard GA Pilo A Olsson M Wahren J. The disposal of an oral glucose load in healthy subjects. a quantitative study. Diabetes. (1985) 34:580–8. 10.2337/diabetes.34.6.580 3891471

[41] Katz LD Glickman MG Rapoport S Ferrannini E DeFronzo RA. Splanchnic and peripheral disposal of oral glucose in man. Diabetes. (1983) 32:675–9. 10.2337/diabetes.32.7.675 6862113

[42] Lin L-Y Kuo H-K Li H-Y Hwang J-J Lin J-W. Confirming a biological pathway in the metabolic syndrome-insight from the NHANES 1999-2002. Obesity. (2008) 16:2676–81. 10.1038/oby.2008.429 18846046

[43] Graham TE Kahn BB. Tissue-specific alterations of glucose transport and molecular mechanisms of intertissue communication in obesity and type 2 diabetes. Horm Metab Res. (2007) 39:717–21. 10.1055/s-2007-985879 17952832

[44] Herman MA Peroni OD Villoria J Schön MR Abumrad NA Blüher M. A novel ChREBP isoform in adipose tissue regulates systemic glucose metabolism. Nature. (2012) 484:333–8. 10.1038/nature10986 22466288 PMC3341994

[45] Hoffstedt J Förster D Löfgren P. Impaired subcutaneous adipocyte lipogenesis is associated with systemic insulin resistance and increased apolipoprotein B/AI ratio in men and women. J Intern Med. (2007) 262:131–9. 10.1111/j.1365-2796.2007.01811.x 17598821

[46] Kursawe R Eszlinger M Narayan D Liu T Bazuine M Cali AMG. Cellularity and adipogenic profile of the abdominal subcutaneous adipose tissue from obese adolescents: association with insulin resistance and hepatic steatosis. Diabetes. (2010) 59:2288–96. 10.2337/db10-0113 20805387 PMC2927952

[47] Roberts R Hodson L Dennis AL Neville MJ Humphreys SM Harnden KE. Markers of *de novo* lipogenesis in adipose tissue: associations with small adipocytes and insulin sensitivity in humans. Diabetologia. (2009) 52:882–90. 10.1007/s00125-009-1300-4 19252892

[48] Virtue S Petkevicius K Moreno-Navarrete JM Jenkins B Hart D Dale M. Peroxisome proliferator-activated receptor γ2 controls the rate of adipose tissue lipid storage and determines metabolic flexibility. Cell Rep. (2018) 24:2005–12.e7. 10.1016/j.celrep.2018.07.063 30134163 PMC6113930

[49] Porter SA Massaro JM Hoffmann U Vasan RS O'Donnel CJ Fox CS. Abdominal subcutaneous adipose tissue: a protective fat depot? Diabetes Care. (2009) 32:1068–75. 10.2337/dc08-2280 19244087 PMC2681034

[50] Stull AJ Galgani JE Johnson WD Cefalu WT. The contribution of race and diabetes status to metabolic flexibility in humans. Metabolism. (2010) 59:1358–64. 10.1016/j.metabol.2009.12.020 20129629 PMC4240223

[51] Kim JY Tfayli H Michaliszyn SF Arslanian S. Impaired lipolysis, diminished fat oxidation, and metabolic inflexibility in obese girls with polycystic ovary syndrome. J Clin Endocrinol Metab. (2018) 103:546–54. 10.1210/jc.2017-01958 29220530 PMC5800835

[52] Schutz Y. The adjustment of energy expenditure and oxidation to energy intake: the role of carbohydrate and fat balance. Int J Obes Relat Metab Disord. (1993) 17(Suppl. 3):S23–7; discussion S41–2. 8124396

[53] Péronnet F Haman F. Low capacity to oxidize fat and body weight. Obes Rev. (2019) 20:1367–83. 10.1111/obr.12910 31353786

[54] Miles-Chan JL Dulloo AG Schutz Y. Fasting substrate oxidation at rest assessed by indirect calorimetry: is prior dietary macronutrient level and composition a confounder? Int J Obes. (2015) 39:1114–7. 10.1038/ijo.2015.29 25771930

