# Supplementary material for: Similar Metabolic Health in Overweight/Obese Individuals With Contrasting Metabolic Flexibility to an Oral Glucose Tolerance Test
Source: Front Nutr. 2021 Nov 16;8:745907. doi: 10.3389/fnut.2021.745907 (PMC8637191; doi:10.3389/fnut.2021.745907)
Supplement: Supplementary file 1 [file Data_Sheet_1.docx]

**Supplementary Figure 1.** Response of respiratory quotient (RQ) and circulating markers to a prolonged fast in individuals with low (Low-MetF) and high (High-MetF) metabolic flexibility. Values were measured before and after a prolonged fast for (A) RQ, (B) insulin, (C) glucose, (D) lactate, (E) non-esterified fatty acids [NEFA], (F) glycerol, (G) triglycerides, and (H) β-hydroxybutyrate [βOHB]. Data are median and interquartile ranges. Data analyzed by repeated-measures ANOVA and Tukey post-hoc.
